# A Comprehensive Review of the Impact of Chromium Picolinate on Testicular Steroidogenesis and Antioxidant Balance

**DOI:** 10.3390/antiox12081572

**Published:** 2023-08-06

**Authors:** Rúben Moreira, Ana D. Martins, Marco G. Alves, Maria de Lourdes Pereira, Pedro F. Oliveira

**Affiliations:** 1 Department of Chemistry, University of Aveiro, 3810-193 Aveiro, Portugal; rubenjesusmoreira@ua.pt (R.M.); cat.mar@ua.pt (A.D.M.); 2 LAQV-REQUIMTE, University of Aveiro, 3810-193 Aveiro, Portugal; 3 Department of Medical Sciences and Institute of Biomedicine (iBiMED), University of Aveiro, 3810-193 Aveiro, Portugal; 4 CICECO-Aveiro Institute of Materials, University of Aveiro, 3810-193 Aveiro, Portugal; mlourdespereira@ua.pt; 5 Department of Medical Sciences, University of Aveiro, 3810-193 Aveiro, Portugal

**Keywords:** chromium picolinate, trivalent chromium, steroidogenesis, reactive oxygen species, antioxidants, testosterone, Leydig cells

## Abstract

Low testosterone (T) levels are a major cause of male infertility, as this hormone is crucial for several processes throughout the entire male reproductive tract. Leydig cells (LC) produce T through testicular steroidogenesis. Disrupted LC function can hinder steroid production and fertility. Among the factors that affect steroidogenesis, endocrine-disrupting chemicals (EDCs) raise concerns, as they disturb hormonal signaling. Chromium is classified as an EDC, and its main forms are hexavalent (Cr(VI)) and trivalent chromium (Cr(III)). While Cr(III) is controversially regarded as an essential metal, its compound Cr(III) picolinate (CrPic_3_) is used as a nutritional supplement due to its antidiabetic and antioxidant properties. This review aims to identify the possible effects of CrPic_3_ on testicular steroidogenesis and thus, on male fertility. The detriments caused by CrPic_3_ in LC include the inhibition of enzymes involved in steroidogenesis, and, as in other cells, the induction of mutagenesis and apoptosis. Remarkably, CrPic_3_ impacts male fertility through the alteration of reactive oxygen species (ROS), T levels, and sperm parameters (sperm motility and abnormal sperm count). However, gaps and inconsistencies exist in the literature concerning its effects on male fertility. Thus, further research is imperative to comprehend the underlying mechanisms of CrPic_3_ in the physiological processes relevant to male fertility, ensuring the supplement’s safety for use by men.

## 1. Introduction

Infertility is the inability to achieve pregnancy after 12 months or more of regular unprotected sexual intercourse [1]. This condition can have male and/or female origins, with the male factor alone being responsible for one-third of all infertility cases, as well as one-half of all combined male-female caused cases [2]. In 2015, it was estimated that 30 million men worldwide were infertile [3]. One of the main contributors to the rise of infertility is the constant exposure to chemical compounds. The effects of many of these chemicals, with which we have daily contact, on the male reproductive system and spermatogenesis remain unknown. Endocrine-disrupting chemicals (EDCs) are a group of compounds considered toxic to humans and to the environment [4,5]. One of the major characteristics of these chemicals is that they interfere with the endocrine signaling of the body [5]. Heavy metals are considered EDCs with known toxicological risk to human health, including to sexual health and male fertility, as they affect several factors, including semen quality parameters and the secretory function of accessory sexual glands [6]. This is not surprising, since 2% of men who suffer from infertility present endocrine disruption as the principal cause [7], and EDCs can disrupt the hypothalamus–pituitary–testis axis [8]. Among the hormonal dysfunctions that can occur, the production of testosterone (T), essential for the normal functioning of the male reproductive system and the triggering of spermatogenesis, is sensitive to exposure to various compounds [9]. A group of several heavy metals is included under the heading of EDCs, with chromium (Cr) being a widely used example across several industries. Among the various Cr compounds, chromium picolinate [tris(picolinate)chromium(III)] (CrPic_3_) has become a very popular supplement [10] to reduce weight or manage blood glucose levels. Although the use of CrPic_3_ has shown some promising positive effects for human health, its safety is up for debate. In addition, the classification of Cr as an essential mineral has been challenged. In this review, we critically discuss the current knowledge concerning the effects of CrPic_3_ on T levels and how exposure and consumption of this heavy metal may impact male fertility through the alteration of reactive oxygen species (ROS).

## 2. Leydig Cells and Testicular Steroidogenesis

**Figure 1:**
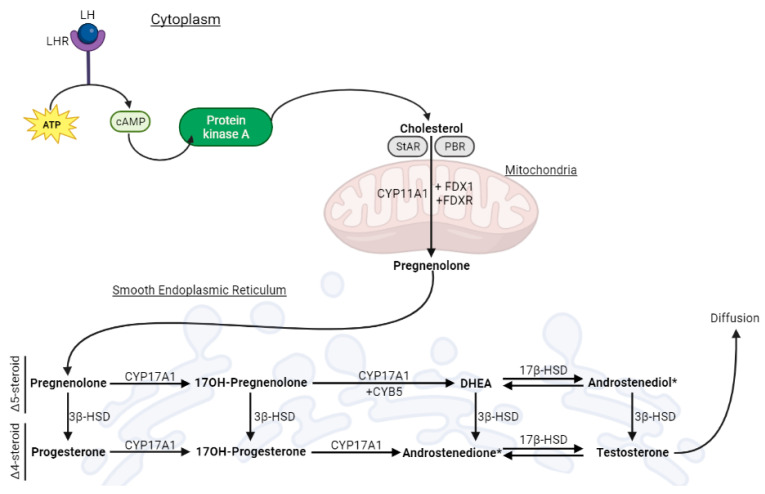
Schematic representation of testicular steroidogenesis that occurs in the Leydig cell. Luteinizing hormone (LH) binds to its receptor (LHR), kicking off the cAMP-PKA (cyclic adenosine monophosphate—protein kinase A) pathway that leads to the release of cholesterol from its cellular reservoirs. Then, cholesterol enters the mitochondria, via the steroidogenic acute regulatory protein (StAR) and the peripheral-type benzodiazepine receptor (PBR), where it is converted to pregnenolone (Preg). Next, pregnenolone is transported to the smooth endoplasmic reticulum, where the remaining steroidogenic reactions occur. The classic pathway yields testosterone as an end-product, which diffuses into the extracellular medium.

The main somatic cells in the testes are Sertoli and Leydig cells (LC) [11]. The latter are found in the connective tissue, between the seminiferous tubules, and produce steroids in a process known as testicular steroidogenesis, summarized in Figure 1. By the end of this process, T is the main steroid produced and will be essential in the regulation of spermatogenesis, the development and maintenance of primary sexual characteristics, which include testicular descent and the growth of the penis and testes, and secondary sexual characteristics, such as the development of male hair patterns and voice deepening [12,13], as well as general androgenic and anabolic effects, like growth during puberty [11,13]. Testosterone exerts its functions by binding to the androgen receptor (AR) in the cytoplasm, allowing it to bind to specific DNA motifs in the nucleus, regulating the transcription of specific genes [14]. In this review, we will focus on the physiology and functions of mature adult LC.

Testicular steroidogenesis that occurs in LC produces 95% of the circulating levels of T that are observed in adult male individuals [15]. It begins when the luteinizing hormone (LH) binds to its receptor (LHR) in the cell membrane [16]. This functions as a signal to a cascade that causes the conversion of ATP into cAMP (cyclic adenosine monophosphate), the activation of protein kinase A (PKA), and the release of cholesterol [16]. Cholesterol is then transported to the mitochondria via a coordinated action of the steroidogenic acute regulatory protein (StAR) and the peripheral-type benzodiazepine receptor (PBR) [17,18]. Afterwards, cholesterol is cleaved by CYP11A1, forming pregnenolone (Preg) [15]. Subsequently, Preg is transferred to the smooth endoplasmic reticulum (SER) [17]. In the SER, Preg is converted into T via either the classic or the backdoor pathway [19]. Herein, we focus the classic pathway, which is known as the main pathway for T synthesis in humans [20]. In this pathway, CYP17A1 plays a pivotal role, and there are two enzymatic activities: 17a-hydroxylase and 17,20-lyase [19]. The 17a-hydroxylase activity causes the conversion of Preg into 17α-hydroxypregnenolone (17OHPreg) and progesterone (Prog) into 17OH-Progesterone (17OHProg). Conversely, the 17,20-lyase activity promotes the conversion of 17OHPreg into dehydroepiandrosterone (DHEA) and 17OHProg into androstenedione. Hydroxysteroid dehydrogenases (HSDs) are also essential to this biosynthesis [19]. 3β-hydroxysteroid dehydrogenase (3β-HSD) catalyzes the conversion of Δ5-steroids (Preg, 17OHPreg, DHEA, and androstenediol) to Δ4-steroids (Prog, 17-OHProg, androstenedione, and T) [19], while 17β-hydroxysteroid dehydrogenase (17β-HSD) catalyzes reversible redox reactions, namely the conversion of DHEA to androstenediol and androstenedione to T [21].

In summary, the classic pathway of steroidogenesis is a multifaceted process that involves a series of enzymes and intermediates. This intricate pathway plays a crucial role in the human male reproductive system, and disturbances in the synthesis of T may result in male subfertility or infertility.

## 3. Chromium: The Good, the Bad, and the Controversial

Cr is the 24th element of the periodic table, and this element can be found in nature in two forms: hexavalent Cr (Cr(VI)) and trivalent Cr (Cr(III)) [22]. Cr(VI) exists as chromate (CrO4-2) or dichromate (Cr2O7-2) ions [22] in an aqueous solution. Cr(III) salts include Cr chloride (CrCl3) and Cr picolinate (Cr(C_6_H_4_NO_2_)_3_) [23]. Both forms exhibit very distinct modes of action and biological effects. Thus, it is imperative to understand their physiological effects and how they are metabolized and excreted from the body. 

### 3.1. Cr(VI): A Toxic Form of Cr

Cr(VI) is used in numerous industries, such as leather tanning, metal processing, and chromate production. Improper management of Cr(VI) may cause ingestion, dermal contact, and/or inhalation by the population, which is known to lead to various health issues [24]. Cr(VI) can enter the cell through non-specific membrane anion transporters. In the cytoplasm, antioxidants such as ascorbate [25], cysteine [26], and reduced glutathione (GSH) reduce Cr(VI), generating reactive oxygen species (ROS) [22] and Cr(III) [27]. As we will discuss, part of the mutagenic effect of Cr(VI) is caused by Cr(III), since it is a by-product of its reduction. The health risks of Cr(VI) include nephrotoxicity [28]; hepatotoxicity [29]; and cancer of the lung, nose, and nasal cavity [30]. There are also well documented toxic effects on the male reproductive system and fertility that include testicular effects, such as loss of testes weight [31]; structural effects, namely moderate tubular necrosis, degeneration of LC [31], and disturbance of the germinal epithelium [32]; and molecular effects, in particular the arrest of spermatogenesis, the decrease in androgenesis [33], and the decrease in antioxidant defenses, such as the levels of superoxide dismutase (SOD) [33]. Cr(VI) is credited as the more hazardous form of Cr, and therefore, it has been the focus of the scientific community for a longer period than has Cr(III). Nevertheless, some studies on Cr(III) are raising concerns regarding its safety [24] and even challenging its classification as an essential element. 

### 3.2. Cr(III): A Controversial Essential Element 

In 1959, Schwarz and Mertz proposed that Cr(III) should be an essential element due to its role in glucose tolerance [34]. Nevertheless, the debate over this classification continues to divide the scientific and clinical communities. An argument in favor of classifying Cr(III) as an essential element is that it is a part of the glucose tolerance factor (GTF), which is synthesized in vivo after the absorption of dietary Cr [35]. GTF is known to bind to insulin, boosting its activity threefold [35]. However, one study found that there was no correlation between blood levels of Cr and glycemic control [36]. Instead, evidence has shown that there are no symptoms of Cr(III) deficiency when there is glycemic dysfunction, thus challenging the criterion used for Cr to be considered essential [37]. Indeed, this suggests that Cr(III) does not fulfill the requirements to be considered an essential element, although it may have medicinal properties [37]. A core topic of discussion regarding this subject is: why should Cr(III) be considered essential, when it is a part of the genotoxicity of Cr(VI)? Maret explained that this could be due to the inability of Cr(III) to enter the cell by itself, since it requires transferrin for entrance, as well as a low-molecular-weight chromium-binding substance (LMWCr) to exert its effects, whereas Cr(VI) can directly enter cells and then be converted to a higher extent to Cr(III). Thus, Cr(III) is more abundant in the cell when it results from Cr(VI) reduction, thereby rendering it more prone to induce adverse effects [10]. 

In 2009, Vincent, who previously considered Cr as essential, published a review to celebrate the 50th anniversary of this classification, stating that it is crucial to identify the biomolecules that form complexes with Cr to understand its biological (side)effects and whether it should actually be considered essential [38]. Even so, since Cr(III) is still considered essential, it is necessary to establish recommended daily doses. In 2014, the European Food Safety Authority (EFSA) determined that the dietary chromium intake should be as follows: 30.1–42.9 μg/day for infants (12 < 36 months), 54.3–71.2 μg/day for children (36 months < 10 years), 63.5–83.4 μg/day for teenagers (10 < 18 years), and 57.3–83.8 μg/day for adults (≥18 years) [39]. However, in 2018, Filippin et al. argued that the daily doses of chromium for adults should be slightly higher, 59.55 μg/day for men and 56.08 μg/day for women, to optimize the nutritional effects and avoid toxicity [40]. Nevertheless, there is still no consensus on the doses of either Cr or CrPic_3_, or more notably, on the safety of Cr(III). This implies that compounds such as CrPic_3_ may not be as safe as previously thought, meaning that more studies supporting its safety or highlighting its toxicological mechanisms should be made available. Future studies will be needed in the years to come to reach a consensus.

## 4. Positive and Adverse Effects of Supplementation with CrPic_3_

CrPic_3_ is composed of Cr(III) chelated with picolinic acid (Pic), with the molecular formula Cr(C_6_H_4_NO_2_)_3_ [41]. Despite the controversy around Cr(III), CrPic_3_ is already abundantly commercialized as a nutritional supplement, particularly targeted to diabetic and obese patients. It is also sold as a supplement to treat depression [42,43], to protect against heat stress [44], to stimulate ovulation in women with polycystic ovary syndrome, and to improve the lipid profile [45], among other announced benefits. 

This section will address the positive effects of CrPic_3_, particularly its antidiabetic and antioxidant properties, as well as its adverse effects, with a special focus on male reproduction and LC.

### 4.1. Antidiabetic Effects of CrPic_3_ and Its Implications on Male Fertility

As previously mentioned, CrPic_3_ is abundantly used as a nutritional supplement because of its antidiabetic properties. Diabetes mellitus is a heterogeneous group of metabolic disorders which includes type 2 diabetes mellitus (T2DM). T2DM is characterized by dysfunction of the insulin-producing pancreatic beta cells, heightened glucagon-producing pancreatic alpha-cell activity, and insulin resistance in the peripheral tissues. When untreated, it leads to hyperglycemia, dyslipidemia, insufficient amino acid uptake, and ATP production [46]

Regarding its antidiabetic effects, CrPic_3_ is capable of: (1) increasing insulin sensitivity [47,48], (2) increasing glucose tolerance and uptake (both basal and insulin-stimulated) [45,49], and (3) preventing damage caused by hyperglycemia [45]. These effects occur because CrPic_3_: (a) decreases the phosphorylation of IRS-1, the JNK pathway [50], as well as pro-inflammatory cytokines, mainly TNFα [51,52], which causes the inhibition of IRS-1 through the phosphorylation of Ser^307^ [53]; (2) increases the presence of the glucose transporter GLUT4 in the cell membrane and stimulates the p38/MAPK pathway [49]; and (3) normalizes the levels of antioxidant enzymes in the liver [45]. CrPic_3_ also assumes a protective role against dyslipidemia, since it improves the altered lipid profile [45], regulates triglycerides and HDL-c [54,55], and decreases serum cholesterol through the increase in SREBP, a transcription factor responsible for cellular cholesterol homeostasis [56]. However, it does not alter the levels of apolipoproteins ApoA and ApoB [57], markers of cardiovascular risk and metabolic syndrome [58]. Martin et al. (2006) showed evidence that in patients with T2D, between 25–75 years old, CrPic_3_ attenuated the increase in body weight, favored body fat distribution, and allowed for the decrease in glucose and increase in insulin sensitivity [47]. With that being said, CrPic_3_ shows promising positive effects regarding diabetes and cardiovascular diseases. However, one must wonder at what cost and if its benefits exceed the possible drawbacks.

Since diabetes mellitus has a strong impact on male fertility, namely a negative effect on sperm parameters (reduced semen volume, count, concentration, and progressive motility) and testosterone levels [59], attempting to ameliorate these effects may be of interest to improve the fertility of men suffering from infertility linked to diabetes. Indeed, Alves et al. found evidence in the literature that led them to believe that, even though the link between diabetes and male infertility is not absolute, there might be mechanisms “of the disease that may affect testicular cells, spermatogenesis, sperm production and sperm maturation” [60]. Interestingly, when Meneses et al. reviewed the effects of metformin, an extensively used antidiabetic drug, on male fertility, they realized that the consensus in the scientific community is that it improves aspects of male fertility, such as levels of FSH, LH, and testosterone; along with sperm concentration, motility, and morphology, among others [61]. This highlights, on one hand, the detrimental effects of diabetes mellitus on male fertility and on the other, the potential benefits of antidiabetic pharmacological substances to ward off those deleterious effects.

### 4.2. Antioxidant Effects of CrPic_3_ in Cellular Systems

A possible positive effect that has been attributed to CrPic_3_ is the increase in antioxidant defenses, which will be explored in this subsection. This is an important feature, since oxidative stress, which results from an imbalance between the production of ROS and antioxidant defense, contributes to the development of numerous pathologies [62]. ROS can, among other effects, disrupt the hormonal crosstalk in the body, causing, for instance, a diminishing of testosterone [63]. Still, it is important to note that the equilibrium of ROS production and elimination is crucial to the functioning of the male reproductive system, since low levels of ROS are required for sperm function, but in excess, ROS cause injury that includes lipid peroxidation and DNA damage [64].

As we will discuss, several antioxidant enzymes are more expressed after various treatments with CrPic_3_, namely GSH, catalase (CAT), SOD, glutathione peroxidase (GPX), and glutathione reductase (GR). Doddigarla et al. found that treating type 2 diabetic rats (high carbohydrate diet induced) orally with 1.4 μg/day of CrPic_3_ for 8 weeks caused a significant increase in GSH and CAT and a significant decrease in MDA [65]. Likewise, Al-Bishri et al. treated type 2 diabetic rats (streptozotocin induced) with 100 μg/kg body weight of CrPic_3_. In doing so, they found that the Cr supplement significantly increased GSH, CAT, GPX, and SOD [66]. Using the same model, Kolahian et al. treated rats orally for 4 weeks with 5 mg/kg of CrPic_3_. This treatment revealed that CrPic_3_ significantly increased CAT, SOD, and GPX and decreased thiobarbituric acid reactive substances (TBARS) [67]. Similarly, Sundaram et al. also studied the antioxidant effects of a 4-week oral treatment with CrPic_3_ (1 mg/kg) in streptozotocin-induced diabetic rats, finding that it significantly increased GR, CAT, SOD, and GSH and decreased MDA [45]. Moreover, Jain et al. and Saiyed and Lugo studied the effects of orally administered CrPic_3_ in human patients from the USA, between 30 to 55 years old, with type 2 diabetes [68,69]. For this purpose, they used 25 (2 men and 23 women) and 43 (sex demographic not disclosed) subjects. In both clinical studies, the subjects were treated orally with 400 μg/day for 3 months [68,69]. While Jain et al. found that CrPic_3_ decreased protein carbonylation in a non-significant manner [68], Saiyed and Lugo, who performed a similar study, but with a larger group of subjects, showed that this decrease is actually significant [69].

SOD is an enzyme responsible for removing the superoxide radical (O^2−^) via its reduction to hydrogen peroxide (H_2_O_2_). In turn, CAT is the enzyme responsible for catalyzing the decomposition of H_2_O_2_ into water and oxygen. Additionally, GPx is also involved in the elimination of H_2_O_2_ by converting GSH into oxidized glutathione (GSSG). Then, GSSG is recycled by a reduction catalyzed by GR, allowing for this cycle to repeat itself [70]. Given these points, it is easy to understand that the significant increase in these enzymes will increase the antioxidant capacity of the cell to protect it from oxidative stress.

The peroxidation of lipids is a consequence of rampant oxidative stress which may cause damage in cells and tissues, since oxidants attack lipids that exhibit carbon–carbon double bonds. These reactions lead to the formation of many products, in particular malondialdehyde (MDA) [71]. Given that MDA is produced as a consequence of oxidative stress, its reduction may indicate a decrease in such stress. Furthermore, the TBARS assay is utilized as a generic test to access lipid peroxidation, since it measures the production of MDA through its reaction with thiobarbituric acid (TBA), which ends with the formation of the conjugate MDA-TBA. Even though this assay has limited analytical specificity, it can demonstrate that MDA is reduced, hence showing that lipid peroxidation is diminished [72]. The previously mentioned studies showed that this is the case in groups treated with CrPic_3_, further leading us to believe that it reduces oxidative stress.

Finally, carbonylation is an irreversible modification caused by ROS and a hallmark of oxidative stress. Protein carbonylation leads to the accumulation of reactive carbonylated species—carbonyl stress—which causes cell death [73]. The decrease in this stress observed in the clinical studies indicates a positive effect of CrPic_3_, preventing the formation of this harmful carbonylated molecule and, in consequence, averting the disruption of the normal physiology of the cell.

All together, these data show that CrPic_3_ appears to exhibit antioxidant effects in cellular systems by enhancing the activity of antioxidant enzymes GSH, CAT, SOD, GPX, and GR, and decreasing lipid peroxidation, and protein carbonylation, thus mitigating damage caused by oxidative stress.

### 4.3. Possible Mechanisms for CrPic3 Toxicity

**Figure 2:**
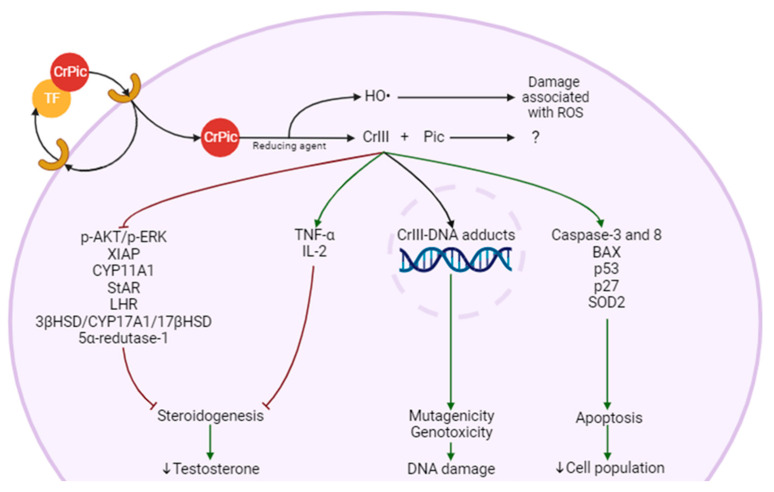
Possible molecular mechanisms of Leydig cell (LC) damage and steroidogenesis impairment from chromium picolinate (CrPic_3_). CrPic_3_ enters the cell bound to transferrin (TF) through endocytosis. In the cell, it is separated from TF due to the low pH of the mature endosome (not shown), and it is reduced to trivalent chromium (Cr(III)) and picolinic acid (PIC), with the production of hydroxyl radical (HO•). Four atoms of Cr(III) bind to chromodulin (not represented) and exert effects such as inhibition of steroidogenesis, DNA damage (also caused by HO•), and cell death.

To our knowledge, no specific mechanism of toxicity has been described for either for Cr(III) alone or CrPic_3_, but there are some reports focused on the effects of these compounds (Figure 2). CrPic_3_ circulates in the blood bound to transferrin [74]. When it binds to its receptor on the cell membrane, it induces the endocytosis of CrPic_3_ [75]. Due to the low pH of the endosome, CrPic_3_ is separated from transferrin and is reduced by an unknown agent that causes the separation into Pic and four Cr(III) atoms and release of ROS [75]. Cr(III) atoms bind to ApoLMWCr, which becomes HoloLMWCr and exert its effects [75]. It is not clear whether Pic is involved in any mechanism of toxicity [76]. In the LC, Cr(III) is reported to be involved in the suppression of LHR, the decline of StAR activity, and the reduction of the level and/or activity of enzymes such as CYP11A1, CYP17A1, 3βHSD, and 17βHSD [77]. These effects have a possible impact on steroidogenesis by decreasing LH signaling, cholesterol entry into the mitochondria, its conversion to Preg and the reactions that occur in the SER. These effects would ultimately affect T production and all the processes that are dependent on this hormone. This also suggests that the mitochondria may be a target of CrPic_3_, since it affects mitochondrial enzymes and, as we will see later, it is involved in stress in this organelle.

Interestingly, CrPic_3_ affects cytokine production in a way that has been described to cause a decrease in IL-6 [51] and TNF-α [51,52]. Contrarily, others report that it increases TNF-α and IL-2 cytokines [78]. This is an important issue, since if pro-inflammatory cytokines are indeed increased, CrPic_3_ could affect steroid production, as these cytokines impact negatively steroidogenesis [79]. Indeed, two authors reported that TNF-α acts as a transcription inhibitor for genes involved in steroidogenesis, such as StAR, with Suescun and collaborators hinting at the possibility of the involvement of the TNFR1 pathway [80,81]. Hales et al. and Wang et al. also highlighted this suppressive behavior of IL-1β and IL-6, respectively, but no pathway has been proposed [81,82]. 

As mentioned earlier, it is suggested that part of the toxicity of Cr(VI) is caused by Cr(III). After Cr(VI) is reduced to Cr(III) in the cell, it enters the nucleus, where it unwinds the DNA and binds to it, forming Cr-DNA adducts [83]. Cr(III) binds to an N7 atom of a guanine, forming two types of complexes in the major grove: binary complexes, comprised of Cr(III) and DNA, or tertiary complexes, with a third molecule [84]. This molecule can be histidine or ascorbate. The histidine complex does not harm the cell [85], while the ascorbate complex crosslinks with DNA, causing strand breaks [25]. Furthermore, the replacement rate of Cr(III)-DNA adducts is low, which means that these can have permanent consequences [84]. 

CrPic_3_ causes signs of apoptosis in the ovarian cell line CHO AA8 when administered at 80 μg/cm^2^, for 48 h [76]. One of these indicators of apoptosis was the mitochondrial swelling and degradation of the cristae in a dose-dependent manner [76]. A recent study has demonstrated that this chromium compound increased the levels of caspase-8 and caspase-3 in the blood of Wistar rats orally treated with 0.3 mg/kg body weight for 8 weeks [78]. Caspase-8 is involved in the extrinsic pathway of apoptosis, while caspase-3 is involved in the intrinsic and extrinsic pathways [86]. In human peripheral blood lymphocytes, a treatment with 50 μM of CrPic_3_ increased expression of caspase 3 by 1.8- and 2.2-fold compared to the control after 24 h and 48 h, respectively. In these cells, 24 h of exposure to 100 μM of CrPic_3_ also increases the BAX/Blc-2 ratio, causes the collapse of the mitochondrial membrane potential, and triggers the displacement of cytochrome c into the cytoplasm, which was 3.2-fold that observed in the control condition. Furthermore, the authors concluded that the cytotoxicity of CrPic_3_ in the lymphocytes is centered around the mitochondria and oxidative stress caused by intracellular ROS [87]. CrPic_3_ also causes apoptosis by increasing BAX in HBL-100 human mammary epithelial cells after treatment with 10 μg/L for 6 h [88]. Additionally, another study used 25 ppm potassium dichromate as a source of Cr(III), rather than CrPic_3_, to study apoptosis in the offspring germ cells of rats after gestational exposure to drinking water containing this chromium compound, from day 9.5 to day 14.5 of gestation [89]. The observed effects included the upregulation of pro-apoptotic cascades p53/p27-BAX-Caspase-3 and p53-SOD2 and a decrease in the expression of anti-apoptotic proteins pAKT, pERK, and XIAP [89]. The p53 tumor suppressor is a pro-apoptotic gene, as well as a protein that is involved in pathways that cause cell death [90]. Regarding p27, it has an ambiguous effect on apoptosis, so it is necessary to clarify if this increase induced by CrPic_3_ is pro- or anti-apoptotic [91]. BAX then causes the release of cytochrome c from the mitochondria [92], which ends up activating caspase-3 [93]. The anti-apoptotic protein pAKT [94] is involved in the phosphoinositide 3-kinase (PI3K)/Akt pathway, which interrupts the progression of the cell cycle [95,96]. pERK and XIAP block apoptosis downstream of the mitochondria, with ERK being activated in phosphorylation by MEK and thus activating XIAP, which inhibits the effector caspases [97]. Hence, Cr(III) appears to have a pro-apoptotic role in the described cells. However, 200 ppb of CrPic_3_ was described to have no negative effect on the proliferation of the myoblast cell line C2C12 after 5 days of exposure, indicating that it is not an inducer of apoptosis in all cell types [98]. All these results suggest that, depending on the conditions of exposure, CrPic_3_ may have pro-apoptotic effects in cells such as ovary cells, lymphocytes, epithelial cells, and germ cells, likely through BAX, as well as caspase-3 and -8. However, these effects are apparently cell-dependent. To our knowledge, no work has been conducted to investigate the possible apoptotic effects of CrPic_3_ on LC.

### 4.4. Impact of CrPic_3_ on Male Fertility

CrPic_3_ has already shown promise as a supplement, with the positive effects mentioned in this review. However, its influence on male fertility is still not fully understood. Nevertheless, in Table 1, we summarize the main effects found in the literature. 

Two studies identified damage in LC after treatment with CrPic_3_ (8 mg and 15 mg/kg body weight for 90 days in albino rats [99] and 0.250, 0.375, and 0.500 mg/animal/day for 84 days in Santa Inês lambs [100]). However, one of these studies suggested that this was an artifact, so more research is needed to clarify these results [100]. Contradictory results were found regarding the effects of CrPic_3_ in LC function. Of four papers that explored this topic, two have reported that the supplement decreases T production, LH levels, and the expression of enzymes involved in steroidogenesis, especially HSDs [99,101]. Contrarily, others state that CrPic_3_ increases T production [55,102]. These contrasting data may be a result of different study designs, which emphasizes the importance of further research to determine the effects on humans.

**Table 1:** Main effects of chromium picolinate (CrPic_3_) on male fertility.

Effects	Dose	Duration	Model	Reference
↑ROS, FSH ↓T, HSD, LH Leydig cell damage Spermatid degeneration	8 and 15 mg/kg body weight	90 days	Male albino rats	[99]
↑T	0.2, 0.4, 0.6, 0.8, 1, or 1.2 mg/kg body weight	84 days	Male Nile tilapia	[55]
↑motility (total and progressive) ↓abnormal sperm	0.009 mg/kg body weight	63 days	Male rabbit bucks	[103]
Retraction of Leydig cells (artifact?)	0.250, 0.375, and 0.500 mg/animal/day	84 days	Santa Inês male lambs	[100]
↑T	0.8, or 1.2 mg/kg body weight	84 days	Matrouh cocks	[102]
↓abnormal sperm	0.08181 mg/kg of food	95 days	Breeding boar	[104]
↓T ↓ reproductive organs weight ↑testicular Cr levels	0.020 mg/0.1 kg body weight	60 days	Wistar male rats	[101]

Abbreviations: FSH—follicle-stimulating hormone; LH—luteinizing hormone; HSD—hydroxysteroid dehydrogenases; ROS—reactive oxygen species; T—testosterone.

Sperm cells may be harmed by CrPic_3_, particularly due to the degeneration of spermatids it causes [99]. It has been hypothesized that this may be due to ROS accumulation; however, we hypothesize that if T levels are indeed reduced (also described in the article), this may cause the detachment of spermatids from the Sertoli cells [105], which would decrease the number of cells that progress to spermatozoa. In spite of this, some sperm parameters are improved after diet supplementation with CrPic_3_, specifically sperm motility and abnormal sperm count. Indeed, the total and progressive motility of spermatozoa was increased in rabbit bucks treated with CrPic_3_ (0.009 mg/kg body weight for 63 days) [103]. Furthermore, abnormal sperm count decreased in rabbit bucks (0.009 mg/kg body weight for 63 days) [103] and breeding boars (0.08181 mg/kg of food) [104].

Taken together, these reports indicate that CrPic_3_ can target both LC and sperm cells, but its action is still far from fully understood.

## 5. Conclusions

In conclusion, as a heavy metal, Cr is classified as an EDC, and it can interfere with endocrine signaling. Cr(III) provokes considerable debate regarding whether it should be considered essential or hazardous. Nevertheless, Cr(III) is used as a nutritional supplement, especially as CrPic_3_, since it exerts antioxidant and antidiabetic, among other, effects. Concerning its effects on male fertility, it appears that CrPic_3_ reduces the expression of a receptor and enzymes involved in steroidogenesis in LC, possibly diminishing T levels. In other types of cells, Cr(III) causes alterations in the production of inflammatory cytokines, particularly TNF-α, mutagenicity, and genotoxicity, and increases apoptosis. If these effects were found in LC, it would affect steroid production and, as a result, male fertility. Further research should include in vitro cytotoxicity studies of LC to determine the cellular consequences of CrPic_3_, along with clinical studies to determine the consequences of this supplement in humans and to assess whether the previously established clinical advantages are worth the risks associated with this Cr(III) compound.
